# Promoting evidence-based health care in Africa

**DOI:** 10.2471/BLT.17.030917

**Published:** 2017-09-01

## Abstract

Countries with major public health challenges do not always base their health-care decisions on the best available scientific evidence. Charles Shey Wiysonge tells Fiona Fleck how he and his colleagues are trying to change that.

Q: How did you become interested in medicine and public health?

A: Growing up in Cameroon I wanted to study mathematics and was drawn to complex equations and aeronautics, but people said I should study medicine, so I did. At medical school most students wanted to become clinical specialists, public health was not popular at all. After graduating, several things drew me to public health. I worked as a hospital physician and saw several outbreaks of measles. People used to say “don’t count your children until measles have come and gone.” Children were dying of measles every day. This experience eventually led me to work on immunization. Also, while working in internal medicine, there were several treatment options for hypertension and heart disease. I was not always sure which was the best. That’s when I discovered evidence-based medicine. At that time – around the year 2000 – the Cochrane Collaboration started a new fellowship to train leaders in evidence-based medicine from low- and middle-income countries. I applied, became the first holder of the award and trained at the Cochrane Centre in Oxford.

Q: What is evidence-based medicine?

A: Research and other evidence are constantly changing and health-care professionals need to keep up with the latest developments so they can offer the interventions that are known to work and not those that are harmful or ineffective. There are many examples of unspeakable and unnecessary suffering resulting from the failure to take an evidence-based approach to clinical care. For example, in 1999 Cochrane researchers did a systematic review to find out whether drugs that inhibited massive calcium influx into cells reduce the risk of death or dependency after stroke. At the time, stroke patients were widely treated with calcium channel blockers. They found no evidence of a beneficial effect. Evidence-based medicine has been defined as the “conscientious, explicit and judicious use of current best evidence in making decisions about the care of individual patients”, although today, health-care professionals prefer the term “evidence-based health care” for a more holistic approach.

“There are many examples of unspeakable and unnecessary suffering resulting from the failure to take an evidence-based approach.”

Q: You did not learn about evidence-based health care at medical school, is it included in medical curricula today?

A: Some medical and dental schools in South Africa offer separate courses on evidence-based health care, others have embedded the approach in all courses, and some do both. Our Cochrane collaborators at the universities of Calabar in Nigeria and Yaoundé I in Cameroon also offer programmes in the evidence-based approach for physicians. Evidence-based health care is included in the curricula for health-care professionals in these and some other African countries, but more needs to be done at the undergraduate and the postgraduate levels across the continent.

Q: The Cochrane Collaboration prepares and disseminates information on what works and what doesn’t in health care globally. How is the South African Cochrane Centre doing this in Africa?

A: Our centre was set up in 1997 as a unit of the South African Medical Research Council. It is the only Cochrane centre in Africa and has a branch in Nigeria. There is increasing recognition in sub-Saharan African countries, that policy-making should be based on the best available scientific evidence. To this end, we have been training researchers in these countries to do systematic reviews and recently expanded these activities via the recently established Cochrane African Network. The Cochrane Collaboration charges a fee to access its global library of systematic reviews and other evidence. Low-income countries can access this evidence via a special online platform, thanks to the Evidence Aid initiative. In South Africa, free access to the Cochrane Library is now provided by the South African Medical Research Council and we hope other middle-income countries in Africa will follow this model.

Q: Are the Cochrane reviews useful in Africa too, or do countries in Africa need evidence that is tailored to their specific situation?

A: Not all Cochrane reviews are applicable to Africa. Since 2007, the South African Cochrane Centre has collaborated with the Centre for Informed Health Choices in Norway and others to improve the use of reliable research evidence in policy and management decisions in Africa. We found, overall, that most primary studies on health system issues are from high-income countries and that only some of these reviews are applicable in low-income settings, including parts of sub-Saharan Africa. For this reason, reviews that synthesize such studies have limited relevance to Africa, especially those written by researchers with no experience in Africa.

Q: So more research is needed that is specific to sub-Saharan Africa?

A: Our analyses suggest that external partners, rather than national priorities, have driven health research in most African countries over the last two decades. However, we don’t need to re-invent the wheel, as much of the global research evidence is applicable to Africa. A major priority for new research in African countries should therefore be implementation research. This type of research helps us understand which health-care interventions work in specific contexts and which do not, and to test different ways to make health-care more effective. African countries often fail to make optimal use of evidence in decision-making, resulting in unnecessary loss of life, reduced quality of life and lost productivity. An example, is acquired immune deficiency syndrome**(AIDS) denialism in South Africa from 1998 to 2003, characterized by the refusal to use antiretrovirals for human immunodeficiency virus (HIV) prevention and treatment. This resulted in a reversal of gains in child survival and life expectancy and the loss of a generation of economically active adults in their prime.

Q: How are you working to make policy-makers in Africa more aware of the need to base their health-care decisions on the best available scientific evidence?

A: The use of health research in decision-making in African countries is generally weak. That’s why we advocate for the dissemination and use of evidence, and building partnerships to promote evidence-informed health care in collaboration with decision makers. African countries sometimes develop new guidelines, but often adopt or adapt existing WHO guidelines to suit national contexts. Our analyses of the development or contextualization of guidelines used in African countries reveals deficiencies in the rigour of development and adherence to international reporting standards. We advise countries on how to develop guidelines for their specific national contexts. We try to work with the health ministries through expert groups to encourage governments to base their policies on the best available evidence and we are involved in initiatives, such as the Effective Health Care Research Consortium, an international group with partners in Cameroon, Kenya, Nigeria and South Africa. However, we are limited by financial constraints, as our work does not attract much funding.

Q: What are the barriers stopping African countries from basing their public health policies and practice on the best scientific evidence?

A: There are several barriers. The health research field in Africa is characterized by numerous players, diverse interests, dispersed efforts and uncertain outcomes in relation to its impact on the major health challenges of the continent. For a better understanding and uptake of scientific evidence in health policy-making, we need a stronger research community – research is not well funded or supported in many African countries – and this community needs to forge closer links with policy-makers, programme managers and implementers. Sometimes the evidence is difficult for policy-makers to understand. That’s why we provide easy-to-read summaries for them. Sometimes, policy-makers ignore the evidence because it’s not what they want to hear. For example, the South African Department of Health once asked the South African Cochrane Centre to prepare a review of the evidence on how best to prevent mother-to-child transmission of HIV infection. But when we gave them the review, they ignored it.

“We have seen a positive change in attitudes towards the use of evidence in health-care planning.”

Q: Have you noticed any improvement in the uptake of evidence in health policy-making?

A: We have seen a positive change in attitudes towards the use of evidence in health-care planning. For example, the Paediatric Association of Kenya has been using explicit, transparent guideline development procedures when developing recommendations for the prevention and treatment of childhood conditions for the last decade. Another example is the increasing number of African countries with functioning national immunization technical advisory groups, which provide evidence-based advice to national health authorities on how to implement WHO vaccine policies and recommendations.

Q: How will the conference you are hosting in Cape Town from 13 to 16 September contribute to improving the uptake of scientific evidence in policy-making in Africa?

A: The Cochrane Collaboration holds a colloquium every year. This year will be the first time that Cochrane will be joined by the four other biggest players in evidence-based policy: the Campbell Collaboration, the Johanna Briggs Institute, the Guidelines International Network and the International Society for Evidence based Health Care, to organise the first ever Global Evidence Summit. We are expecting thousands of participants, including those from every health-care discipline and from virtually every country on the continent of Africa. There is strong evidence that such interactive, educational meetings, can help health-care workers improve their performance. Participants will have the chance to learn more about guideline development, using evidence for emerging global health and social crises and how the evidence community can overcome denial of clear scientific findings. We are really excited about this opportunity to highlight the evidence-based approach to policy and practice in health for countries in Africa.

